# Vascular Ehlers-Danlos Syndrome: A Comprehensive Natural History Study in a Dutch National Cohort of 142 Patients

**DOI:** 10.1161/CIRCGEN.122.003978

**Published:** 2024-04-16

**Authors:** Serwet Demirdas, Lisa M. van den Bersselaar, Rosan Lechner, Jessica Bos, Suzanne I.M. Alsters, Marieke J.H. Baars, Annette F. Baas, Özlem Baysal, Saskia N. van der Crabben, Eelco Dulfer, Noor A.A. Giesbertz, Apollonia T.J.M. Helderman-van den Enden, Yvonne Hilhorst-Hofstee, Marlies J.E. Kempers, Fenne L. Komdeur, Bart Loeys, Daniëlle Majoor-Krakauer, Charlotte W. Ockeloen, Eline Overwater, Peter J. van Tintelen, Marsha Voorendt, Vivian de Waard, Alessandra Maugeri, Hennie T. Brüggenwirth, Ingrid M.B.H. van de Laar, Arjan C. Houweling

**Affiliations:** Department of Clinical Genetics, Cardiovascular Institute, Erasmus Medical Center, University Medical Center, Rotterdam, the Netherlands (S.D., L.M.v.d.B., R.L., D.M.-K., H.T.B., I.M.B.H.v.d.L.).; European Reference Network ReCONNET, Ehlers Danlos Syndrome Working Group, Rotterdam, the Netherlands (S.D.).; Department of Human Genetics, Amsterdam University Medical Center, Vrije Universiteit Amsterdam, the Netherlands (J.B., S.I.M.A., M.J.H.B., S.N.v.d.C., F.L.K., E.O., A.M., A.C.H.).; Department of Human Genetics, Amsterdam University Medical Center, University of Amsterdam, the Netherlands (J.B., S.I.M.A., M.J.H.B., S.N.v.d.C., F.L.K., E.O., A.C.H.).; Department of Genetics, University Medical Center Utrecht, the Netherlands (A.F.B., N.A.A.G., P.J.v.T.).; Department of Human Genetics, Radboud University Nijmegen Medical Center, the Netherlands (O.B., M.J.E.K., B.L., C.W.O., M.V.).; Department of Genetics, University Medical Center Groningen, the Netherlands (E.D., E.O.).; Clinical Genetics, Maastricht University Medical Center, the Netherlands (A.T.J.M.H.-v.d.E.).; Department of Clinical Genetics, Leiden University Medical Center, the Netherlands (Y.H.-H.).; Department of Medical Biochemistry, Amsterdam University Medical Center, Amsterdam Cardiovascular Sciences, the Netherlands (V.d.W.).; European Reference Network for Rare Multisystemic Vascular Disease, Medium Sized Arteries Working Group, Rotterdam, the Netherlands (I.M.B.H.v.d.L.).

**Keywords:** aortic aneurysm, connective tissue, collagen, Ehlers-Danlos syndrome, type IV, uterine rupture

## Abstract

**BACKGROUND:**

Vascular Ehlers-Danlos syndrome (vEDS) is a rare connective tissue disorder with a high risk for arterial, bowel, and uterine rupture, caused by heterozygous pathogenic variants in *COL3A1*. The aim of this cohort study is to provide further insights into the natural history of vEDS and describe genotype-phenotype correlations in a Dutch multicenter cohort to optimize patient care and increase awareness of the disease.

**METHODS:**

Individuals with vEDS throughout the Netherlands were included. The phenotype was charted by retrospective analysis of molecular and clinical data, combined with a one-time physical examination.

**RESULTS:**

A total of 142 individuals (50% female) participated the study, including 46 index patients (32%). The overall median age at genetic diagnosis was 41.0 years. More than half of the index patients (54.3%) and relatives (53.1%) had a physical appearance highly suggestive of vEDS. In these individuals, major events were not more frequent (*P*=0.90), but occurred at a younger age (*P*=0.01). A major event occurred more often and at a younger age in men compared with women (*P*<0.001 and *P*=0.004, respectively). Aortic aneurysms (*P*=0.003) and pneumothoraces (*P*=0.029) were more frequent in men. Aortic dissection was more frequent in individuals with a *COL3A1* variant in the first quarter of the collagen helical domain (*P*=0.03).

**CONCLUSIONS:**

Male sex, type and location of the *COL3A1* variant, and physical appearance highly suggestive of vEDS are risk factors for the occurrence and early age of onset of major events. This national multicenter cohort study of Dutch individuals with vEDS provides a valuable basis for improving guidelines for the diagnosing, follow-up, and treatment of individuals with vEDS.

Heterozygous pathogenic variants in the *COL3A1* gene (OMIM 120180), encoding for type III collagen, cause the heritable connective tissue disorder vascular Ehlers-Danlos syndrome (vEDS; OMIM 1300050, ORPHA 286)^1^ with an estimated prevalence range from 1:50 000 to 1:200 000 in the United States.^2^ Spontaneous arterial dissections, bowel rupture, and uterine rupture are the most characteristic and life-threatening complications of vEDS.^3,4^ At age 40 to 43 years, a first vascular, gastrointestinal, or obstetric complication has occurred in 80% to 85% of the individuals.^1,4^ Initially, the median survival for individuals with vEDS was 48 to 51 years, with a higher number of deaths due to arterial dissection or rupture in adolescent males.^4,5^ Patients with a phenotype classically associated with the disease are reported to have characteristic appearance/features including prominent (sunken) eyes, narrow nose, gingival recession/fragility, lobeless ears, acrogeria, easy bruising, translucent skin, and early-onset varicose veins.^6^ Revised suggestive major and minor criteria for vEDS have been published in 2017.^7^ These revised criteria were created to indicate when genetic testing is recommended. The presence of one of the following major criteria was proposed as an indication for genetic testing: (1) positive family history of vEDS, (2) arterial rupture or dissection under 40 years of age, (3) spontaneous sigmoid colon perforation, (4) unexplained uterine rupture in the third trimester of pregnancy, or (5) spontaneous carotid-cavernous sinus fistula formation. Genetic testing should also be considered in the presence of a combination of the following minor criteria: characteristic appearance as mentioned above, spontaneous pneumothorax, talipes equinovarus, congenital hip dislocation, hypermobility, keratoconus, and tendon and muscle rupture.^7^

*COL3A1* is located on chromosome 2q32.2 and consists of 51 coding exons that encode a 1466 amino acid protein.^8^ More than half of the known pathogenic variants in *COL3A1* are missense substitutions of a glycine in the repeating (Gly-X-Y) sequence within the triple helical region of type III collagen. Together with splice variants resulting in exon skipping and in-frame insertions, deletions, and duplications, these variants with a dominant negative effect comprise the majority of the pathogenic variants in *COL3A1*.^1,4^ Variants leading to haploinsufficiency are more rare and may result in a milder phenotype with reduced penetrance compared with dominant negative variants.^1,5,9^ Bi-allelic *COL3A1* variants have been found in <1% of all vEDS individuals.^10,11^

Rarely specific heterozygous arginine-to-cysteine substitutions in *COL1A1* cause a vEDS-like phenotype.^12^

Surveillance in vEDS is very difficult due to the unpredictability of the occurrence of arterial dissections, often without a prior aneurysm,^2,4^ and the potential complexity of surgical interventions.^13^ Although recommendations for patient management have been proposed,^3,14,15^ these are often based on observations in case reports or small cohorts due to the rarity of the disease. It is therefore essential to gain further insight into the natural history of the disorder by detailed studies in larger patient cohorts. To this end, we describe a detailed overview of the Dutch cohort of 142 patients with vEDS.

## METHODS

The data that support the findings of this study are available from the corresponding author upon reasonable request. The full methods of this retrospective study are available in Supplemental Data, including Tables S1 and S2. This study was approved by the Medical and Ethics Review Committee of the Amsterdam University Medical Centre (MEC 2019-0662) and all participating centers. The alive participating individuals provided written informed consent before inclusion in the study.

## RESULTS

### Clinical Characteristics of the Study Population

In total, 142 individuals (50% female) from 70 families were included (Table 1). Forty-six of the participants (32.4%) were index patients. Fifteen individuals were deceased, including 10 index patients. The median age at death was 43.0 years (interquartile range [IQR], 23.75–56.50) for index patients, compared with 65.0 years (IQR, 51.5–72.5) for relatives. Six index patients and 2 relatives died due to vascular complications of vEDS. The cause of death was unknown for 3 individuals, including 2 index patients (40 and 61 years) and 1 relative (61 years). All 3 individuals with an unknown cause of death suffered from a major event earlier in life. Six individuals were deceased before vEDS was diagnosed. The median age at genetic diagnosis was 41.0 years (IQR, 28.0–59.5) and 40.0 years (IQR, 29.75–54.5) for index patients. The median time in follow-up at the time of inclusion in the study was 3.0 years (IQR, 1.0–6.0). The main reason for referral for index patients was an aneurysm or dissection (65.2%), and 7 index patients (14.9%) had a facial appearance or other features consistent with vEDS. A total of 34/96 (35.4%) of the relatives with a pathogenic/likely pathogenic (P/LP) variant in *COL3A1* had a major event.

**Table 1.**
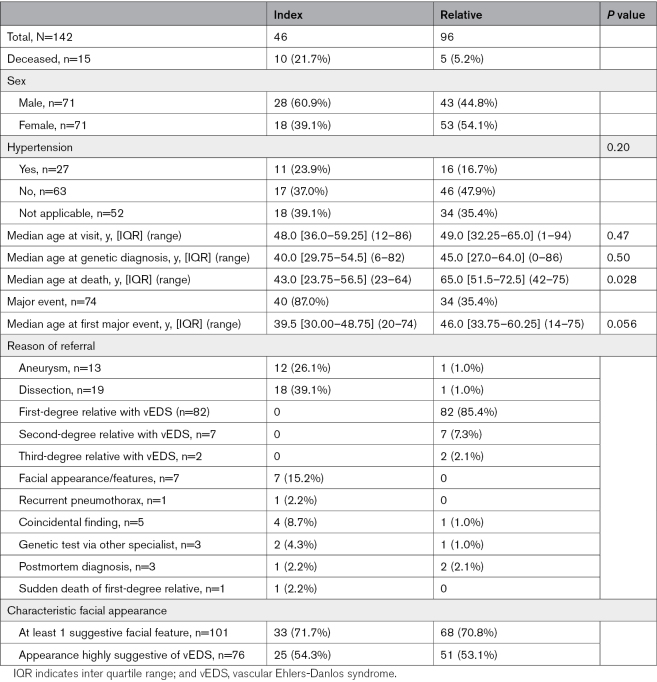
Clinical Characteristics

### 2017 Suggestive Criteria

Almost two-thirds (31/46) of the index patients met the 2017 suggestive criteria for vEDS; 17 of them had at least 1 major criterion, while 14 had a combination of minor criteria. Arterial rupture or dissection was the most common major suggestive criterion in index patients (76.5%). At least 1 major suggestive criterion was present in 75.4% of all individuals, including 37% of the index patients. Another major suggestive criterion besides the “positive family history for vEDS” criterion was present in 9 of 90 relatives (10%), including an arterial rupture or dissection <40 years in 4, a colon rupture in 4, and spontaneous carotid-cavernous sinus fistula formation in 1. Six index patients did not meet any of the major or minor suggestive criteria, including 3 with an isolated aortic aneurysm, 2 diagnosed due to an incidental finding while performing DNA analysis for another condition (age at inclusion, 15 and 61 years), and 1 with an isolated iliac artery dissection at 41 years of age. The individuals with isolated aortic aneurysms were a 63-year-old male with a 45-mm ascending aortic aneurysm (height 184 cm, weight 84 kg), a 56-year-old female who underwent a personalized external aortic root support procedure for a 53-mm aortic root aneurysm (*Z*-score 8.44), and a 69-year-old male who underwent an endovascular aortic repair procedure because of a 64-mm abdominal aortic aneurysm (height 171 cm, weight 77.8 kg). In total, 101 of 124 examined individuals (81.5%) had at least 1 characteristic facial feature, including 76 individuals with >4 facial features.

### Hypertension

One of the risk factors for arterial aneurysms and dissections is hypertension. Data on blood pressure were known for 94 individuals (66.2%), and 27 of 94 (28.7%) had hypertension.

Antihypertensive drugs were prescribed in 74 of 142 (52.1%) individuals, including the 27 individuals with hypertension and 47 without hypertension who received antihypertensive drugs because of the diagnosis of vEDS. Twenty-two individuals used >1 antihypertensive drug. A beta blocker was prescribed in 61 of 74 individuals (82.4%), including celiprolol in 37 of 74 individuals (50.0%) and other beta blockers in 24 of 74 individuals (32.4%). Thirteen individuals (17.6%) received other antihypertensive drugs: calcium channel blockers in 7, angiotensin-converting enzyme (ACE) inhibitors in 3, angiotensin receptor blockers (ARB) in 2, and an ACE inhibitor plus a calcium channel blocker in 1.

### Children

In total, 8 children (age <18; 6%) were included, of whom 3 were index patients (age range, 12–16 years). The median age at genetic diagnosis and inclusion was 4.5 years (IQR, 0.25–9.00) and 8.0 years (IQR, 3.50–12.75), respectively. Five children underwent genetic testing because 1 of their parents was diagnosed with vEDS, 2 because of facial appearance or features consistent with vEDS, and 1 underwent a genetic test for intellectual disability (a de novo deletion of the complete *COL3A1* gene). None of these children were diagnosed with a major event (age range at inclusion, 1–16 years). In the total cohort, 2 individuals had a major event under the age of 18 years. Most children (5/8) received yearly follow-up by a pediatric cardiologist using different imaging modalities.

### Molecular Studies

**Figure 1.**
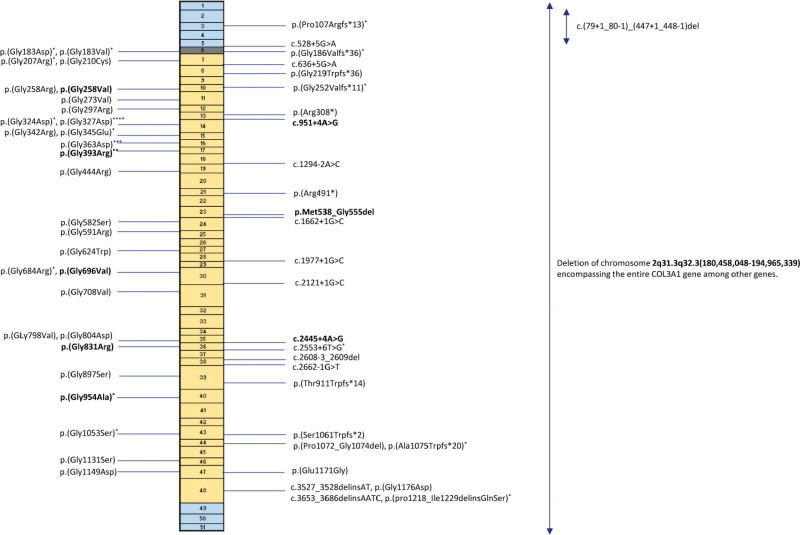
**Schematic overview of the *COL3A1* gene, with the location of pathogenic and likely pathogenic variants found in our cohort.** The boxes represent the coding exons and the colors represent protein domains. Blue boxes represent N and C terminal domains, gray represents the transitional domain, and yellow represents the collagen helical domain. Glycine substitutions are displayed on the left. Each * is an individual with an aortic dissection. Variants in bold are proven de novo variants.

A total of 56 different P/LP *COL3A1* variants were detected, including 29 glycine substitutions, 10 splice variants, 8 frameshift variants, 2 deletion-insertion (delins) variants, 2 nonsense variants, 3 in-frame deletions, 1 glutamate substitution, and 1 copy number variation of 14.5 Mb encompassing the entire *COL3A1* gene, among others (Figure 1; Table S3). In total, 43 of 56 (76.8%) of the detected variants in *COL3A1* are predicted to have a dominant negative effect. Forty-three variants were novel. More than half of the individuals were heterozygous for a glycine substitution, including 63% of the index patients. Nine variants (27%) were found in >1 family, and 9 variants (27%) were proven de novo. The origin of 23 variants was unknown as parental testing could not be performed. Segregation information was available for 39 families, and in total, 94 of 243 (38.7%) first-degree relatives were screened for the familial *COL3A1* variant, revealing 54 heterozygotes, including 23 with a major event in their medical history.

### First Major Event

**Figure 2.**
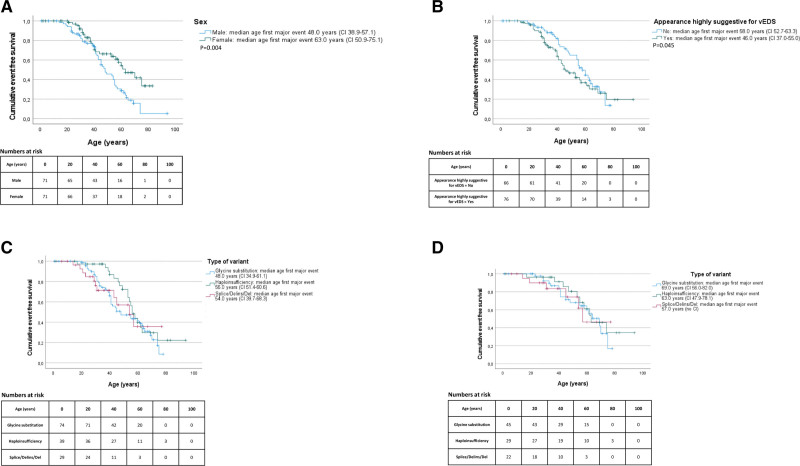
**Kaplan-Meier curves for occurrence of major events. A**, Sex and age at occurrence of the first major event. Age at the first major complication correlated with sex in 142 individuals with vEDS. **B**, Facial features highly suggestive of vEDS and age at occurrence of the first major event. Age at the first major complication correlated with the presence of ≥5 suggestive features of vEDS upon physical examination in 142 individuals with vEDS. **C**, Age at occurrence of the first major event in the total cohort. Age at the first major complication correlated with the type of P/LP *COL3A1* variant in 142 individuals with vEDS. **D**, Age at occurrence of the first major event in relatives. Age at the first major complication correlated with the type of P/LP *COL3A1* variant in 96 relatives with vEDS. P/LP indicates pathogenic/likely pathogenic; splice/delins/del, splice-site/deletion insertion/deletion variant; and vEDS, vascular Ehlers-Danlos syndrome.

In total, 74 individuals (52%) experienced a major event, including 44 individuals with a glycine substitution, 11 with a splice/delins/del variant, and 19 with a predicted haploinsufficient variant. The first major event was lethal in 5 individuals with an aortic dissection, including 4 index patients (2 type A, 1 type B dissection, and 1 abdominal aortic dissection) and 1 relative (unknown origin of dissection). In all, 5 vEDS were diagnosed postmortem. The overall median age at the first major event was 41.0 years (IQR, 31.0–55.0). Significantly more men compared with women suffered from a major event (48 versus 26; odds ratio [OR], 3.61 [95% CI, 1.82–7.14]; *P*<0.001) and the Kaplan-Meier analysis showed a significantly younger age at the time of the first major event in men compared with women (*P*=0.004; Figure 2A).

The youngest individual with a major event was a male who suffered from a myocardial infarction at the age of 14 years due to an aneurysm combined with narrowing up to 70% of the left anterior descending artery. He was 24 years old at the time of inclusion and heterozygous for a pathogenic splice variant in *COL3A1* with a predicted dominant negative effect. A major event was present in 17 of 27 individuals (63.0%) with hypertension compared with 27 of 63 (42.9%) without hypertension (OR, 2.27 [95% CI, 0.98–2.21]; *P*=0.08).

### Major Event and Appearance Highly Suggestive of vEDS

An appearance highly suggestive of vEDS was defined as the presence of 5 or more suggestive features of vEDS upon physical examination and was seen in 76 individuals (53.5%), equally divided in index patients (54.3%) and relatives (53.1%; Table 1). Individuals with an appearance highly suggestive of vEDS did not suffer from a major event more frequently than those without (OR, 1.05 [95% CI, 0.54–2.02]; *P*=0.89). However, the median age at the first major event was significantly lower in individuals with an appearance highly suggestive of vEDS compared with those without: 39.5 years (IQR, 29.0–45.75) versus 48.5 years (IQR, 37.5–60.0), respectively (*P*=0.01). There was no confounding relationship between an appearance highly suggestive of vEDS and type of variant in *COL3A1* (*P*=0.14). Also, the Kaplan-Meier analysis showed a significantly younger age at the first event in individuals with an appearance highly suggestive of vEDS compared with those without (*P*=0.045; Figure 2B).

### Vascular Complications

Sixty-eight individuals were diagnosed with an arterial aneurysm or dissection at a median age of 44.0 years (IQR, 37–55), including 39 index patients (57.4%; Table 2). Twenty-eight individuals (41.2%) had >1 aneurysm or dissection; the majority (22/28) were index patients. Aortic aneurysms were found in 32 of 68 individuals, including 8 individuals with aneurysms/dissections elsewhere and 13 individuals with an ascending aortic aneurysm (Table S4). Aortic aneurysms were significantly more frequent in men than in women (24 versus 8; OR, 3.73 [95% CI, 1.52–9.17]; *P*=0.003). The youngest individual with an aortic aneurysm was 19 years old. No significant difference in the frequency of aortic dissections was seen between men (n=15) and women (n=8; OR, 1.94 [95% CI, 0.76–5.00]; *P*=0.16). The youngest age at which an aortic dissection occurred was 23 years. More than half of the aneurysms in middle-sized arteries (MSAs) were observed in the common and external iliac arteries (61%). Dissections occurred most frequently in the carotid arteries and the common and external iliac arteries (both 37%). The youngest age at which a dissection of a MSA occurred was 16 years. Five individuals (3 male and 2 female) had a spontaneous coronary artery dissection at a mean age of 39.0 years (±3.5). It was the first major event in 3 of them. Three of these individuals had vascular complications elsewhere as well. One individual with a spontaneous coronary artery dissection was an index patient without other phenotypic characteristics highly suggestive of vEDS, as reported by Bos et al.^16^

**Table 2.**
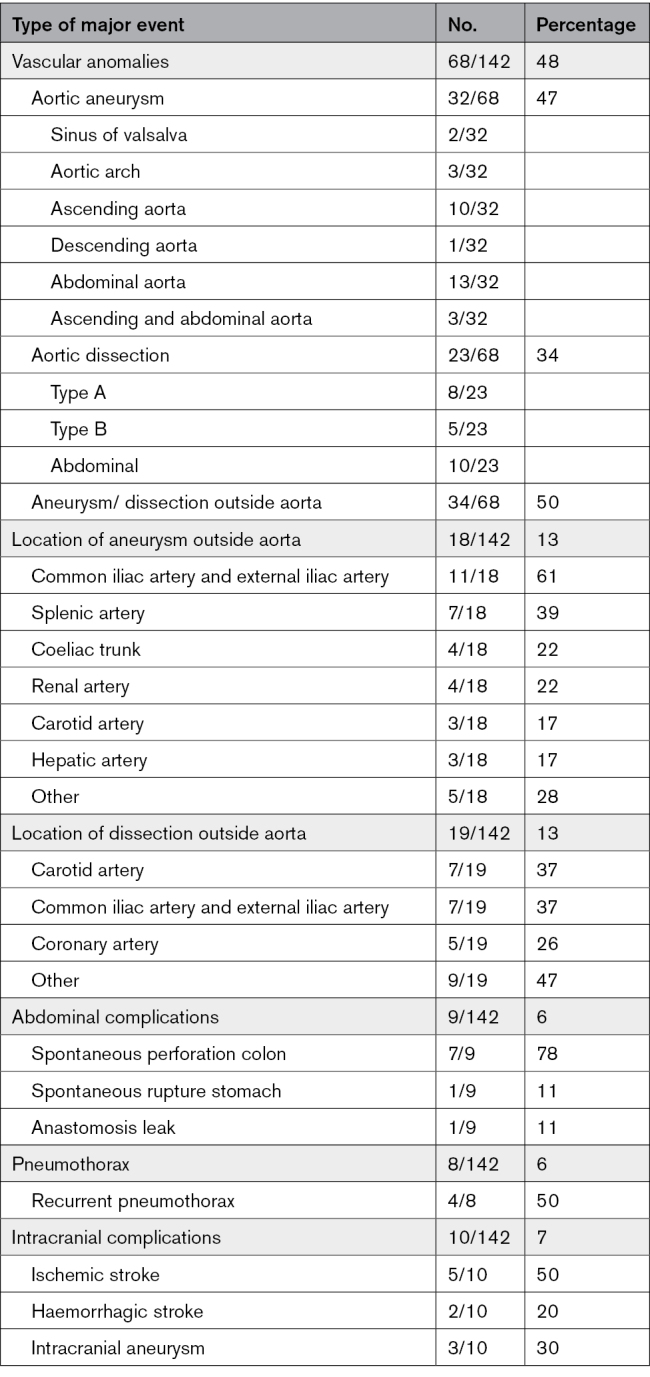
Major Events in Individuals With Vascular Ehlers-Danlos Syndrome

Forty-five surgical interventions were performed for vascular complications in 33 individuals (48.5%; Table S5). Severe complications were noted in 4 individuals, including a sternum infection in 1, a burst abdomen with enterocuteneous fistulas in 1, a type 3 endoleak resulting in an abdominal aortic aneurysm rupture leading to a serosa defect with ileocecal resection in 1, and an arteria hepatica rupture after coiling in 1.

### Abdominal and Urogenital Complications

Major abdominal complications occurred in 9 individuals (7 male and 2 female), including spontaneous colon perforation in 7 (median age 32.0 years [IQR, 30.0–44.0]), 1 spontaneous stomach rupture (age unknown; male), and 1 anastomosis leak after colectomy (age 74 years; male; Table 2). The 2 women with spontaneous colon perforation were 31 years old (splice variant) and 27 years old (glycine substitution). Both had highly suggestive features for vEDS upon physical examination. Five of the spontaneous colon perforations (71.4%) were located in the sigmoid or rectum. Because of the spontaneous colon perforations, hemicolectomy or partial colectomy was performed in 5. Complications due to the surgery occurred in 2, namely an anastomosis leak plus an enterocutaneous fistula in 1 and a rupture of the spleen in the other individual.

In total, 129 pregnancies were noted, and no uterine ruptures were observed in our cohort.

### Spontaneous Pneumothorax

Eight individuals suffered from spontaneous pneumothorax, including recurrent spontaneous pneumothorax in half of them (Table 2). Pneumothorax occurred significantly more frequently in men compared with women, namely 7 versus 1 (OR, 7.69 [95% CI, 0.92–62.5]; *P*=0.029). The median age at first spontaneous pneumothorax was 21.5 years (IQR, 17.25–29.0), and the youngest was 16 years. The diagnosis of vEDS was highly suspected in 2 individuals due to the presence of suggestive features of vEDS upon physical examination and vascular abnormalities in the MSAs.

Spontaneous pneumothorax was the first presentation of vEDS in 4, whereas vascular complications occurred later between the ages of 23 and 50 years. Two individuals (age 42 and 62 years) had recurrent pneumothorax as adolescents without other phenotypic characteristics for vEDS.

### Cerebrovascular Complications

In total, 7 individuals (4 male and 3 female) endured a spontaneous stroke at a median age of 49.0 years (IQR, 45.0–58.0; 5 ischemic and 2 hemorrhagic strokes; Table 2). The youngest individual was a 22-year-old male with a spontaneous subdural hematoma. Ischemic stroke occurred once due to bilateral internal carotid artery dissection and once due to a vertebral artery dissection and of unknown etiology in the other 3.

Presymptomatic imaging of the cerebral arteries was performed in 58 of 142 individuals with vEDS, and 3 (5.2%) were diagnosed with an intracranial aneurysm. Carotid-cavernous fistula was diagnosed in 2 individuals (3.4%). Furthermore, 4 ischemic strokes occurred as complications during/after vascular surgery.

### Genotype-Phenotype Correlation

#### Glycine Substitutions

The median age at the first major event for individuals with a glycine substitution and individuals with a splice/delins/del variant was significantly lower compared with individuals with a suspected haploinsufficient variant, namely, 40.5 years (IQR, 31.0–53.25) and 30.0 years (IQR, 21.0–45.0) versus 53.0 years (IQR, 43.0–58.0; *P*=0.027 and *P*=0.002).

The Kaplan-Meier analysis showed a significantly lower age at the first major event in individuals with a glycine substitution in *COL3A1* compared with individuals with a suspected haploinsufficient variant (*P*=0.044; Figure 2C). Individuals with a splice/delins/del variant in *COL3A1* were slightly younger at the time of their first major event compared with individuals with a suspected haploinsufficient variant (*P*=0.06; Figure 2C). Surprisingly, when taking only relatives into account, no difference in age at the first major event was seen between the different variant categories (*P*=0.62; Figure 2D).

Aortic dissections seem to be more present in individuals with a glycine substitution (23.0%) compared with suspected haploinsufficient variants (10.3%) or splice/delins/del variants (6.9%; *P*=0.09; Table 3). There was no difference in the frequency of aortic aneurysms, aneurysms, or dissections outside the aorta between the variant categories (*P*=0.60 and *P*=0.370, respectively). However, 4 of 5 individuals with spontaneous coronary artery dissection were heterozygous for a glycine substitution, including 2 individuals from the same family. About half of the individuals with a colon perforation (55.6%) had a glycine substitution in *COL3A1.* Only 1 individual had a variant predicted to result in haploinsufficiency, namely a 43-year-old male with a spontaneous sigmoid perforation. More than half of the individuals with pneumothorax (62.5%) or cerebral complications (57.1%) were heterozygous for a glycine substitution.

**Table 3.**
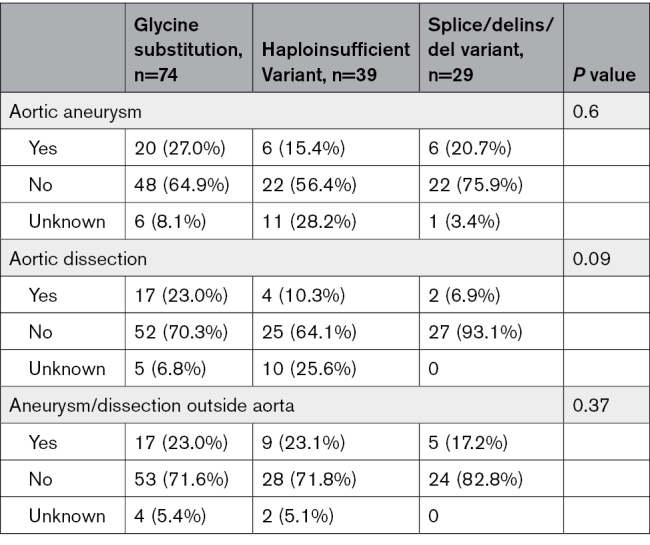
Vascular Complications and Genotype

#### Localization

Interestingly, we found that individuals with an aortic dissection had significantly more often a P/LP variant located in the first quarter of the collagen helical domain of *COL3A1* than in the other parts of the collagen helical domain, namely 13 of 20 (65.0%) versus 7 of 20 (35.0%; *P*=0.03) for unrelated individuals and 16 of 23 (69.6%) versus 7 of 23 (30.4%; *P*=0.01) when taking all individuals with an aortic dissection into account (Figure 1). There was no correlation between bowel complications, pneumothorax, or strokes and the type of location of the variant in the collagen helical domain in *COL3A1*.

### Surveillance

At the time of inclusion, 111 of 127 living individuals (87.4%) received periodic medical follow*up, including annual medical follow-up in more than half (55%). Medical follow-up was mainly provided by the (vascular) internist, cardiologist, or vascular surgeon (Table 4). The majority (73.9%) had arterial imaging at least every other year. A high degree of variability was seen in the imaging modalities used, including magnetic resonance angiography, computed tomography angiography, and echocardiography (Table 4). Sixteen individuals did not receive follow-up for several reasons (Table 4).

**Table 4.**
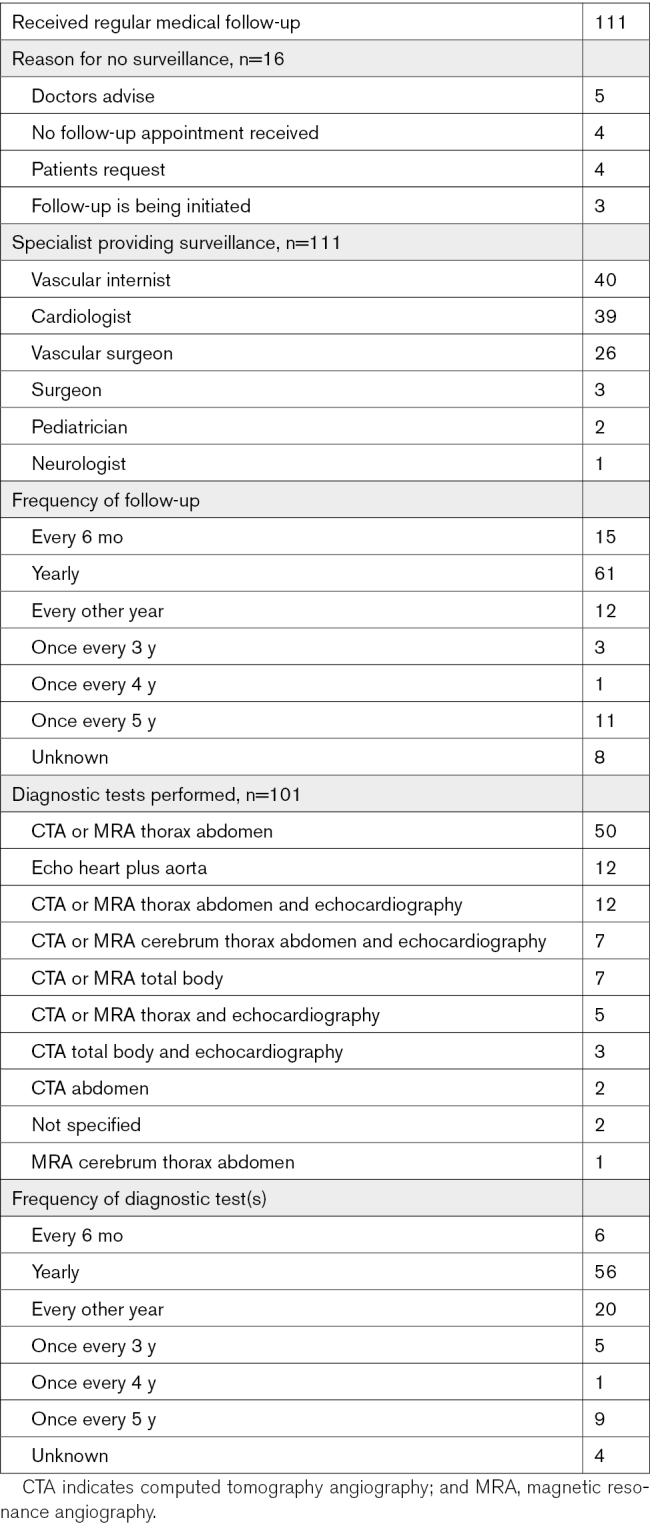
Surveillance of Individuals With Vascular Ehlers-Danlos Syndrome

## DISCUSSION

In this Dutch national multicenter natural history study, we provide a detailed overview of the phenotype and genotype of 142 individuals from 70 families, harboring 56 different P/LP variants in *COL3A1*. Genetic examination revealed a glycine substitution in the *COL3A1* gene in almost two-thirds (63%) of the index patients, which corresponds with the current literature^1,5^ and the Ehlers-Danlos Syndrome Variant Database (https://databases.lovd.nl/shared/variants/COL3A1). In index patients, the diagnosis of vEDS is most of the time made after a complication of vEDS. In this study, we describe the natural history of vEDS until diagnosis and the clinical evolution of the disease afterwards.

More than half of the individuals suffered from one or more major events. Male sex (*P*<0.001) and glycine substitutions (*P*=0.045) seem to give a higher risk for major events. Moreover, individuals with a glycine substitution were significantly younger at the time of the first major event compared with those with a suspected haploinsufficient variant (*P*=0.044), which is in line with several previous reports.^1,5,9,17^ Interestingly, when solely looking at the relatives in our cohort, this difference in age at the first major event between the different variant categories is not observed (*P*=0.62). We speculate that this might be due to the fact that relatives start with arterial screening and management of their blood pressure at a younger age or due to the presence of unknown genetic modifiers. In addition, vEDS individuals with hypertension seem to be younger at the time of the first major event compared with vEDS individuals without hypertension (*P*=0.08); therefore, adequate management of blood pressure is highly recommended.

Furthermore, major events were not more frequent in individuals with an appearance highly suggestive of vEDS (*P*=0.90); however, they did have a significantly lower median age at the time of their first major event compared with those without an appearance highly suggestive of vEDS (*P*=0.01). An appearance highly suggestive of vEDS was defined as the presence of more than half of the 9 mentioned features, so 5 or more. There were no differences in the predictive ability of individual features. Additionally, how obvious these features were varied between individuals, which can be challenging for the caregiver. The correlation between the incidence of major events and an appearance highly suggestive of vEDS has not been described before in vEDS. A correlation between the severity of craniofacial features and their cardiovascular outcome has also been described in individuals diagnosed with Loeys-Dietz syndrome.^18,19^ Caregivers should be aware of this increased risk in these individuals.

We can only speculate on the factors involved in this correlation, since no proposed mechanism has been reported to date. Various features of the appearance of individuals with vEDS are likely to be explained by the expression of *COL3A1* in the following tissues: (1) smooth muscle cells (varicose veins), (2) skin (easy bruising, translucent skin, gingival regression/fragility, lobeless ears, and acrogeria) and (3) bone marrow plus connective tissue (prominent [sunken] eyes and narrow nose; https://www.proteinatlas.org/ENSG00000168542-COL3A1/tissue). The presence of the abovementioned features might give an indication of the impact of the *COL3A1* variant on an individual. When more features are present, the *COL3A1* variant might have a greater impact on that individual, including a more severe effect on the hollow organs and blood vessels. Additionally, the great phenotypic variability even within families might implicate a role for cofounders. These cofounders likely differ per individual and might result in a different impact of the *COL3A1* variant. Furthermore, the type of variant does not explain this correlation, since an appearance highly suggestive of vEDS was equally present in individuals with a dominant negative variant (55/103, 53.4%) and a haploinsufficient variant (21/39, 53.8%).

None of the 8 children in our cohort have had a major event, and only 2 individuals in the total cohort suffered from a major event as a child. Major events have repeatedly been described in childhood.^1,5,20^ The low number of children in our cohort causes a bias in the described age at the first major event and the prevalence of major events in children. An explanation for the low number of children is the restraint in presymptomatic genetic testing in children in the Netherlands. Genetic testing for vEDS is offered to children with an affected first-degree relative, but the majority of parents refrain from genetic testing in childhood. Dutch parents and clinicians generally attach great importance to the child’s autonomy and therefore prefer presymptomatic genetic testing at an age when their child can make his or her own decision.

Aortic dissections occurred mostly in individuals with a P/LP variant in the first quarter of the collagen helical domain of the *COL3A1* gene. The extracellular matrix of the aortic wall consists primarily of elastin and collagen to provide strength to the aortic wall, to withstand the forces of blood pressure.^21–23^ The arterial collagen is mainly types I and III collagen.^24^ Previous studies in the skin biopsies of patients vEDS revealed an association between collagen fibril diameter and the location of the P/LP variant in *COL3A1*.^25,26^ P/LP variants near the N terminus were associated with larger collagen fibril diameters, whereas mutations near the C terminus of the *COL3A1* gene were associated with smaller collagen fibril diameters. Lapiere et al^27^ showed a higher collagen I/III ratio in fibrils with larger collagen fibril thickness, which suggests reduced presence/incorporation of collagen type III when the P/LP variant is located near the N terminus (the start of the gene). We observed that vEDS individuals with aortic dissection mostly have a P/LP variant in the first quarter of the *COL3A1* collagen helical domain, which is near the N terminus; thus, a shift in the collagen I/III ratio is expected to cause a more severe vascular phenotype. To the best of our knowledge, this genotype-phenotype correlation has not been described previously.

Interestingly, *COL3A1*-deficient, haploinsufficient, and glycine-mutant vEDS mouse models have been reported to show similar features as vEDS patients.^28–32^ Overexpression of *COL3A1* with a variant at the N terminus showed reduced collagen in the aortic adventitia with fibrils highly variable and increased in diameter, similar to those found in the deficiency models. Upon fibril analysis, a higher collagen I/III ratio in fibrils with larger collagen fibril thickness was found in the skin of these *COL3A1* mice.

To date, MSAs are described as the main location of vascular complications of vEDS.^1,3,4^ However, in our cohort, aneurysms or dissections occurred more frequently in the thoracic and abdominal aortas as compared with the MSA (55 versus 31, respectively). This is likely partially due to ascertainment bias, since the *COL3A1* gene is present in the gene panel for thoracic aortic aneurysms and dissections and analyzed in all individuals with thoracic aortic aneurysms and dissections (n≥1000) who underwent genetic testing. Nevertheless, when taking only relatives into account, the aorta was still more prone to aneurysms or dissections compared with the MSA (23 versus 12).

However, not all individuals (101/127, 79.5%) received imaging at the time of inclusion in the study, and imaging of the abdominal arteries was not performed in 17 of 101 individuals (16.8%). Since vascular complications of the MSA are most commonly present in the abdominal arteries and not all individuals have received imaging of this section (yet), this might explain the underrepresentation of vascular aneurysms in the MSA in our cohort.

In our cohort, 33% of the index patients did not meet the revised criteria for vEDS, which is lower than the 45% previously reported by Ritelli et al,^33^ which suggests that the revised criteria might be too strict.^33^ The wide availability of genetic testing allows for a relatively low threshold for genetic testing. It is suggested to offer genetic analysis including the *COL3A1* gene in all individuals with a dissection or unexplained aneurysm under the age of 50 years, or 60 years in the absence of hypertension.^34^ A previous study on the diagnostic yield of the thoracic aortic aneurysms and dissections genes showed a P/LP *COL3A1* variant in 6 of 810 individuals (0.7%), including 2 individuals in the age range of 50 to 60 years.^35^

Spontaneous pneumothorax and abdominal complications were less frequently present than observed previously (12.5%–17% and 21%–22.6%, respectively).^1,14,36,37^ These complications occurred at a younger age than vascular complications and can be an early manifestation of vEDS.^37^ The median age at the time of a colon rupture or perforation in our cohort was higher (32.0 years [IQR, 30.0–44.0]) than described before by Frank et al^1^ (23.0 years [range, 19–34]). We recommend health care providers to be aware of vEDS in young individuals with spontaneous pneumothorax or abdominal complications, even in the absence of other vEDS features, and at least perform a physical examination and evaluate the family history for features suggestive of vEDS, but also Marfan syndrome and Birt-Hogg-Dubé syndrome, which are also associated with an increased risk for primary spontaneous pneumothorax.^38,39^ Genetic testing should be considered if additional physical or other features or a positive family history are present.

Most of the major abdominal complications (7/9) affected the colon and occurred in individuals with a glycine substitution or splice/delins/del variant in *COL3A1*.^1^ However, we also documented an abdominal complication in an individual heterozygous for a predicted haploinsufficient variant in *COL3A1*, which has only been reported once.^17^

Twelve individuals (8.5%) were diagnosed with intracranial vascular anomalies, including 2 individuals with an ischemic stroke due to the dissection of an internal carotid or vertebral artery, which is in line with the prevalence of intracranial vascular complications described by Adham et al.^40^ The prevalence of carotid-cavernous fistula in our cohort (2.4%) is lower than previously reported (9.8%).^40,41^ Despite the occurrence of intracranial vascular complications in 8.5% of vEDS individuals in our cohort, only 70% of the alive individuals received cerebrovascular imaging. We recommend to consider cerebrovascular screening at least once for all adult individuals with vEDS.

The described phenotypic spectrum of vEDS and genotype-phenotype correlations are based on the data of 142 individuals with vEDS. Since not all individuals with vEDS agreed to participate in this study and some were diagnosed after the closure of the study, the number of known individuals with vEDS in the Netherlands is currently estimated at ≈200. Given the total of 17.4 million inhabitants of the Netherlands, the minimal estimated prevalence of vEDS in the Netherlands is around 1.1:100 000, assuming all patients with vEDS have currently been diagnosed. This estimated prevalence is in line with the previously estimated prevalence range of 1:50 000 to 1:200 000 in the United States.^2^ However, it is likely that prevalence is even higher since the diagnosis can be missed due to the absence of the suggestive criteria for vEDS or unawareness of the suggestive criteria with the health professional.

Assessment of the type and frequency of surveillance revealed a great variability in surveillance strategies of caregivers. Around 70% of the 127 living individuals received surveillance including imaging at least every other year. Different imaging modalities are used, but magnetic resonance angiography or computed tomography angiography of the thorax and abdomen were the most common. This is in line with the observation that arterial monitoring of adult clinically silent patients with vEDS is standard clinical practice in European expert centers of the MSA working group of the European Reference Network For Rare Vascular Diseases.^42^ At this moment, there are no international guidelines for surveillance in vEDS. The objective of surveillance is the prevention of complications by performing elective procedures when abnormalities are present. However, no criteria for elective interventions in vEDS have been clearly established, probably due to the high risk of complications.^1,2,14,17^ Byers et al^3^ recommended to provide a care team for each vEDS patient with a primary physician to coordinate the management and surveillance. Previous studies examined the effect of celiprolol on the prevention of vascular complications and improving survival.^1,43^ Both studies showed a decrease in vascular complications and a higher survival rate in individuals who received celiprolol.

However, both studies had multiple limitations in study design and inclusion criteria, and therefore, no clear conclusions about the effect of celiprolol can be made at this moment.^44^

Given the high variability in type and frequency of surveillance and even the absence of medical follow-up in some vEDS patients, it is important to create an international consensus statement for the management and surveillance of individuals with vEDS. The collaboration of vEDS expert teams is essential in the creation of such a consensus statement, which should be created by using the merged genotypic and phenotypic data of different vEDS cohorts from all over the world.

## CONCLUSIONS

Male sex, the phenotypic appearance, and the type and location of the variant are risk factors for the occurrence and age of onset of a major event. The type of *COL3A1* variant does not influence the age at the first major event in relatives. Caregivers should be aware of the potential diagnosis of vEDS in patients with an isolated major event, even in the absence of characteristic features highly suggestive of vEDS given the phenotypic variability. Because of the great variability in type and frequency of surveillance in current daily practice, it is important to create guidelines for the management and surveillance of individuals with vEDS.

### Limitations

This Dutch cohort of individuals with vEDS is relatively large given the rarity of vEDS. However, the number of included individuals is still small to interpret the results and draw conclusions. Therefore, it would be very valuable for further research to merge all the described vEDS cohorts and perform a meta-analysis. In addition, estimation of the penetrance of rare genetic disorders in retrospective cohort studies has several limitations, including missing data and the likelihood of preferential testing in individuals and relatives affected by complications (eg, inclusion bias). It is therefore not surprising that the penetrance of pathogenic variants in population databases is lower than that observed in index patients and their relatives opting for genetic testing, as recently underlined by Forrest et al.^45^ However, since these observations are representative of families opting for genetic testing (eg, the population reported in our current study), they are highly relevant. Nevertheless, it is important to consider these limitations.

Another common limitation of retrospective cohort studies for such rare diseases is the inclusion of family members, which might pose a risk for dependent data. We assumed that the variability in the vEDS phenotype is mutually independent, even within families.

Only a few children are included in this study due to the reluctance of presymptomatic genetic testing in children in the Netherlands. The low number of children results in a higher median age of the studied cohort. Additionally, this cohort study is limited by the lack of a unified strategy for imaging and follow-up of individuals with vEDS. For example, vascular complications in the MSA were underrepresented in our cohort. This may be explained by the fact that asymptomatic vascular complications of the MSA are most commonly present in the abdominal arteries, and imaging of the abdominal MSA was not performed in all individuals.

## ARTICLE INFORMATION

### Acknowledgments

The authors thank all the research participants, physicians, genetic counsellors, and investigators who have included individuals in this study and collected clinical data.

### Sources of Funding

None.

### Disclosures

None.

### Supplemental Material

Supplemental Methods

Tables S1–S5

References 1,4,5,34,46–57

## Supplementary Material


