# Supplementary material for: Vascular Ehlers-Danlos Syndrome: A Comprehensive Natural History Study in a Dutch National Cohort of 142 Patients
Source: Circ Genom Precis Med. 2024 Apr 16;17(3):e003978. doi: 10.1161/CIRCGEN.122.003978 (PMC11188628; doi:10.1161/CIRCGEN.122.003978)
Supplement: Supplementary file 1 [file hcg-17-e003978-s001.pdf]

SUPPLEMENTAL MATERIAL

Supplemental Methods

Study population

Index patients and relatives (both children and adults) with a P/LP *COL3A1* variant or copy number variation (CNV) from eight academic clinical genetic centres in the Netherlands were included (Supplementary table 1). Children with an affected first degree relative were considered eligible for genetic testing for the familial COL3A1 variant. There is no age restriction for presymptomatic genetic testing for vEDS in the Netherlands. However, there is a reluctance for presymptomatic genetic testing for vEDS in Dutch parents and healthcare providers, causing low numbers of children to be diagnosed with vEDS. The reluctance is due to potential insurance issues, the unclear benefits of establishing the diagnosis in childhood and vascular screening is generally not performed in young children.
Retrospective collection and detailed analysis of molecular and clinical data was performed. Cross-sectional assessment of enrolled patients with a onetime visit at the outpatient clinic was performed between 2019 and 2021 to complete the clinical data and systematically chart the phenotype of index patients and, if available, first/second degree affected family members carrying the familial *COL3A1* variant. All data were entered in a pseudo-anonymized database available only for the researchers.

Measurements of clinical features

Absolute aortic diameters of ≥40 mm for the thoracic aorta and ≥30 mm for the abdominal aorta were classified as an aortic aneurysm [34, 46]. An arterial aneurysm of a middle sized artery was defined as a 50% increase in the normal diameter of the vessel [47]. The z-score of a Sinus of Valsalva aneurysm was calculated with a web-based calculation tool on the Marfan foundation site (https://marfan.org/dx/z-score-adults/), that uses the normograms of Devereux et al [48] and the method of Dubois and Dubois [49] to calculate the BSA. Hypertension was defined as a blood pressure above 90/140 mm Hg and/or the need for antihypertensive drugs [50]. Individuals without hypertension who preventively received an antihypertensive drug because of the diagnosis vEDS were defined as no hypertension. Major events were defined as death due to complications of vEDS, arterial aneurysm or dissection, bowel rupture, other organ rupture, spontaneous pneumothorax or stroke.
Suggestive features of vEDS upon physical examination were considered: prominent (sunken) eyes, narrow nose, gingival recession/fragility, lobeless ears, acrogeria, easy bruising, translucent skin, early onset varicose veins (<30 years of age and nulliparous if female) and clubfeet. An appearance highly suggestive for vEDS was defined as the presence of five or more of these suggestive features

(Supplementary table 2). Clinical illustrations of the above mentioned features are available on https://elementsofmorphology.nih.gov/.

Variant analysis

The pathogenicity of variants in the *COL3A1* gene (NM_000090.3, NG_007404.1) was assessed using Alamut (Alamut Visual Plus, version 1.5.1, 2021 Sophia Genetics), integrating data from large-scale population genetic studies, evolutionary conservation of nucleotides and amino acids, *in silico* missense and mRNA splicing prediction tools. *COL3A1* variants with a minor allele frequency (MAF) > 1% in the Genome Aggregation Database were excluded from further analysis. If possible, co-segregation of variants with the disease phenotype was analyzed. Variants were interpreted according to the 2015 American College of Medical Genetics and Genomics/Association of Molecular Pathology (ACMG/AMP) guidelines [51]. Only variants classified as P/LP were included in this study. In the case of splice variants, if fibroblasts were available, RNA sequencing was performed to investigate the effect on splicing. Nonsense variants and frameshift variants were classified as most likely leading to haploinsufficiency. The first quarter of the collagen helical domain of the *COL3A1* gene was defined as the first ten exons of this domain.

Statistical analysis

Because of small patient numbers much of the data has a skewed distribution. Continuous variables were summarized by median and interquartile range. Categorical data are expressed in frequencies and percentages. Continuous data were compared using the Wilcoxon Mann-Whitney U test or the Kruskal-Wallis tests. Comparisons in categorical data were made using the chi-square test or Fisher exact test. To prevent bias from dependent data, only one member (if available the index patient) per family was included in the analysis of the associations between major events and location of the variant in *COL3A1*. Comparisons of major events and the location of the variants in *COL3A1* were made for all major events separately. Associations of time until the first major event were examined using a Kaplan-Meier curve. Because early major events weigh more heavily than major events at older age, the Gehan-Breslow-Wilcoxon test was used to assess the significance. All tests are 2-sided and significance was assumed at p-value < 0.05. Variables with a p-value <0.05 in univariate analysis were eligible for the binary logistic regression model. Odd ratios, confidence intervals and p-values were used for interpretation. Statistical analysis was performed using SPSS software version 22 (IBM Corp. Released 2013. IBM SPSS Statistics for Windows, Version 22.0. Armonk, NY, USA).

Supplemental Table 1. Inclusion procedure

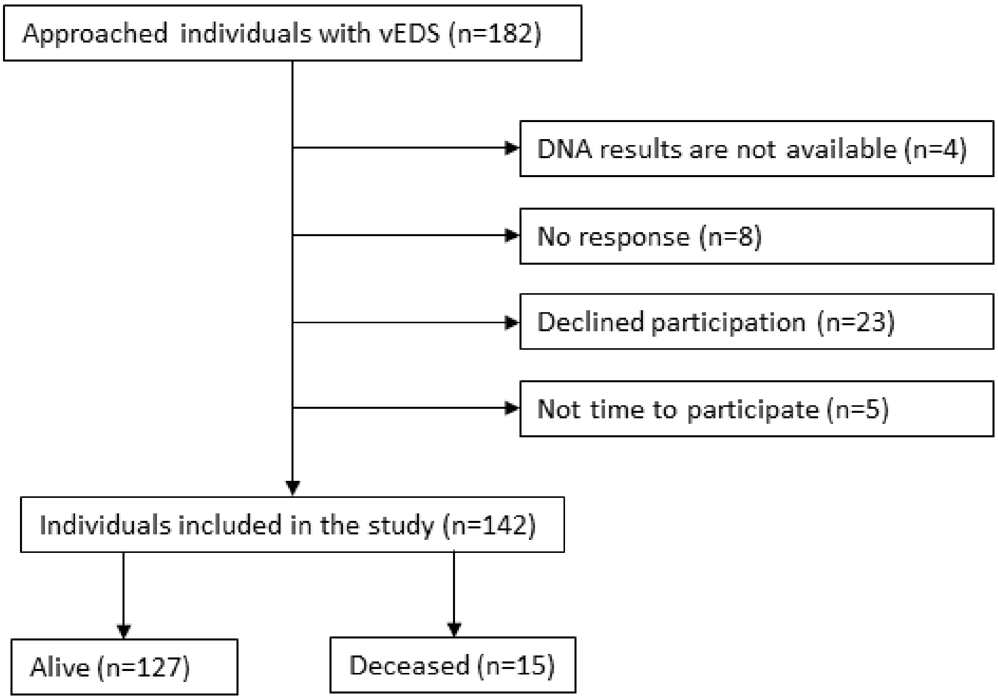

Supplemental Table 2. Criteria for an appearance highly suggestive for vEDS

**Feature**	**Yes = 1 points/ no = 0 points**
Prominent (sunken) eyes	
Narrow nose	
Gingival recession/fragility	
Lobeless ears	
Acrogeria	
Easy bruising	
Translucent skin	
Early onset varicose veins (<30 years of age and nulliparous if female)	
Clubfeet (one sided or bilateral)	
**Appearance highly suggestive for vEDS**	**≥5 points**

Supplemental Table 3. Overview of identified pathogenic and likely pathogenic *COL3A1* variants

Location	Nucleotide change	RNA and/or protein change	Coding effect	Variant category	MAF gnomAD	Pathogenic effect predicted (in silico)[Ɨ]	Splice site prediction	Evidence[#ΘΔT~]	Classification	De novo	Ref
	c.(79+1_80-1)_(447+1_448-1)del	p.(?)	Frameshift	Haploinsufficient*	Absent	-	-	PVS1 PS4 PM2(s)	Pathogenic	Unknown	
Exon 3	c.318_325del	p.(Pro107Argfs*13)	Frameshift	Haploinsufficient	Absent	-	-	PVS1 PS4 PM2(s)	Pathogenic	Unknown	
Intron 5	c.528+5G>A	r.0<br>p.(0)	Splice	Haploinsufficient*	Absent	-	-72.7%	PS4 PM2(s) PP1 PP5 PP3	Likely pathogenic	No	(1)
Exon 6	c.548G>A	p.(Gly183Asp)	Missense	Glycine substitution	Absent	3/4	-	PS4 PM1 PM2(s) PM5 PP2 PP3 PP5	Pathogenic	Unknown	(1, 4, 5)
Exon 6	c.548G>T	p.(Gly183Val)	Missense	Glycine substitution	Absent	3/4	-	PS4 PM1 PM2(s) PM5 PP2 PP3 PP5	Pathogenic	Unknown	(5, 52)
Exon 6	c.555del	p.(Gly186Valfs*36)	Frameshift	Haploinsufficient	Absent	-	-	PVS1 PS4 PM2(s) PP5 PP1	Pathogenic	No	(52)
Exon 7	c.619G>A	p.(Gly207Arg)	Missense	Glycine substitution	Absent	4/4	-	PS4 PM1 PM2(s) PM5 PP2 PP3	Pathogenic	Unknown	(52)
Exon 7	c.628G>T	p.(Gly210Cys)	Missense	Glycine substitution	Absent	4/4	-	PS4 PM1 PM2(s) PP2 PP3	Likely pathogenic	Unknown	
Intron 7	c.636+5G>A	r.584_636+1del<br>p.(Gly195_Ser212del)	Splice	Splice/delins/del	Absent	-	- 64.4%	PS4 PM2(s) PP1 PP5 PP3	Likely pathogenic	No	(4, 53)
Exon 8	c.654dupT	p.(Gly219Trpfs*17)	Frameshift	Haploinsufficient	Absent	-	-	PVS1 PS4 PM2(s) PP1	Pathogenic	No	
Exon 10	c.753del	p.(Gly252Valfs*11)	Frameshift	Haploinsufficient	Absent	-	-	PVS1 PS4 PM2(s) PP5 PP1	Pathogenic	Unknown	(52)
Exon 10	c.772G>A	p.(Gly258Arg)	Missense	Glycine substitution	Absent	4/4	-	PS4 PM1 PM2(s) PM5 PP2 PP3	Pathogenic	Unknown	(5, 52)
Exon 10	c.773G>T	p.(Gly258Val)	Missense	Glycine substitution	Absent	3/4	-	PS4 PM1 PM2(s) PM5 PM6 PP2 PP3	Pathogenic	Yes	(52)

Exon 11	c.818G>T	p.(Gly273Val)	Missense	Glycine substitution	Absent	3/4	-	PS4 PM1 PM2(s) PP2 PP3	Likely pathogenic	Unknown	
Exon 12	c.889G>A	p.(Gly297Arg)	Missense	Glycine substitution	Absent	4/4	-	PS4 PM1 PM2(s) PP2 PP3 PP5	Pathogenic	No	(52)
Exon 13	c.922C>T	p.(Arg308*)	Nonsense	Haploinsufficient	Absent			PVS1 PS4 PM2(s)	Pathogenic	Unknown	
Intron 13	c.951+4A>G	r.898_951del p.(Gly300_Ala317del)	Splice	Splice/delins/del	Absent	-	-65.0%	PS4 PM2(s) PM6 PP5	Likely pathogenic	Yes	(1)
Exon 14	c.971G>A	p.(Gly324Asp)	Missense	Glycine substitution	Absent	3/4	-	PS4 PM1 PM2(s) PM5 PP2 PP3	Pathogenic	Unknown	(52)
Exon 14	c.980G>A	p.(Gly327Asp)	Missense	Glycine substitution	Absent	3/4	-	PS4 PM1 PM2(s) PP1 PP2 PP3 PP5	Pathogenic	No	(52)
Exon 15	c.1024G>A	p.(Gly342Arg)	Missense	Glycine substitution	Absent	4/4	-	PS4 PM1 PM2(s) PP2 PP3 PP5	Pathogenic	No	(52)
Exon 15	c.1034G>A	p.(Gly345Glu)	Missense	Glycine substitution	Absent	3/4	-	PS4 PM1 PM2(s) PP1 PP2 PP3	Pathogenic	No	
Exon 16	c.1088G>A	p.(Gly363Asp)	Missense	Glycine substitution	Absent	3/4	-	PS4 PM1 PM2(s) PP1 PP2 PP3	Pathogenic	No	
Exon 17	c.1177G>C	p.(Gly393Arg)	Missense	Glycine substitution	Absent	4/4	-	PS4 PM1 PM2(s) PM6 PP2 PP3	Pathogenic	Yes	
Intron 18	c.1294-2A>C	p.(?)	Splice	Splice/delins/del*	Absent	-	-100%	PVS1(m) PS4 PM2(s)	Likely pathogenic	Unknown	(1)
Exon 19	c.1330G>A	p.(Gly444Arg)	Missense	Glycine substitution	Absent	4/4	-	PS4 PM1 PM2(s) PP2 PP3 PP5	Pathogenic	Unknown	(4)
Exon 21	c.1471C>T	p.(Arg491*)	Nonsense	Haploinsufficient	Absent	-	-	PVS1 PS4 PM2(s) PP5 PP1	Pathogenic	No	(54)
Exon 23	c.1609-47_1662+58del	p.(Met538_Gly555del)	In frame del	Splice/delins/del	Absent	-	-100%	PVS1(m) PS4 PM2(s) PM4 PM6	Pathogenic	Yes	

Intron 23	c.1662+1G>C	p.(?)	In frame del	Splice/delins/del*	Absent	-	-100%	PVS1(m) PS4 PM2(s) PM4 PP5	Pathogenic	No	(1, 4)
Exon 24	c.1744G>A	p.(Gly582Ser)	Missense	Glycine substitution	Absent	4/4	-	PS4 PM1 PM2(s) PP1 PP2 PP3 PP5	Pathogenic	Unknown	(1, 4, 5)
Exon 25	c.1771G>C	p.(Gly591Arg)	Missense	Glycine substitution	Absent	4/4	-	PS4 PM1 PM2(S) PM5 PP2 PP3	Pathogenic	No	(5)
Exon 27	c.1870G>T	p.(Gly624Trp)	Missense	Glycine substitution	Absent	4/4	-13,8%	PS4 PM1 PM2(s) PP2 PP3	Likely pathogenic	Unknown	
Intron 28	c.1977+1G>C	r.1924_1977del p.(Gly642_Pro659del)	Splice	Splice/delins/del	Absent		-100%	PS4, PM1, PM2(s) PP3	Likely pathogenic	No	
Exon 30	c.2050G>A	p.(Gly684Arg)	Missense	Glycine substitution	Absent	4/4	-	PS4 PM1 PM2(s) PP2 PP3 PP5	Pathogenic	No	(52)
Exon 30	c.2087G>T	p.(Gly696Val)	Missense	Glycine substitution	Absent	4/4	-	PS4 PM1 PM2(s) PM5 PM6 PP2 PP3	Pathogenic	Yes	(5)
Intron 30	c.2121+1G>C	p.(Gly675_Lys707del)^{V}	Splice	Splice/delins/del	Absent	-	-100%	PVS1 PS4 PM2(s) PP5	Pathogenic	Unknown	(5)
Exon 31	c.2123G>T	p.(Gly708Val)	Missense	Glycine substitution	Absent	4/4	-	PS4 PM1 PM2(s) PP1 PP2 PP3 PP5	Pathogenic	No	(5)
Exon 35	c.2393G>T	p.(Gly798Val)	Missense	Glycine substitution	Absent	4/4	-	PS4 PM1 PM2(s) PP2 PP3	Likely pathogenic	Unknown	
Exon 35	c.2411G>A	p.(Gly804Asp)	Missense	Glycine substitution	Absent	4/4	-	PS4 PM1 PM2(s) PP1 PP2 PP3	Pathogenic	No	
Intron 35	c.2445+4A>G	p.(?)	Splice	Splice/delins/del*	Absent	-	-52.2%	PS4 PM2(s) PM6 PP3	Likely pathogenic	Yes	
Exon 36	c.2491G>C	p.(Gly831Arg)	Missense	Glycine substitution	Absent	4/4	-	PS4 PM1 PM2(s) PM6 PP2 PP3	Pathogenic	Yes	
Intron 36	c.2553+6T>G	p.(Gly816_Ala851del)	Splice	Splice/delins/del	Absent	-	-29.9%	PS4 PM2(s) PP1 PP3	Likely pathogenic	No	

Exon 38	c.2608-3_2609del	r.[2608_2661del, 0] p.[Gly870_Asn887del, 0]	Splice	Splice/delins/del	Absent	-	-100%	PS4 PM2(s) PP3	Likely pathogenic	No	
Intron 38	c.2662-1G>T	p.(?)	Splice	Haploinsufficient	Absent	-	-100%	PVS1(m) PS4 PM2(s) PP1	Likely pathogenic	No	
Exon 39	c.2689G>A	p.(Gly897Ser)	Missense	Glycine substitution	1/249220	4/4	-	PS4 PM1 PM2(s) PP1 PP2 PP3 PP5	Pathogenic	Unknown	(52)
Exon 39	c.2731_2732del	p.(Thr911Trpfs*14)	Frameshift	Haploinsufficient	Absent			PVS1 PS4 PM2(s)	Pathogenic	No	
Exon 40	c.2861G>C	p.(Gly954Ala)	Missense	Glycine substitution	Absent	4/4		PS4 PM1 PM2(s) PM5 PM6 PP2 PP3	Pathogenic	Yes	(5)
Exon 43	c.3157G>A	p.(Gly1053Ser)	Missense	Glycine substitution	Absent	4/4		PS4 PM1 PM2(S) PM5 PP2 PP3	Pathogenic	Unknown	(5)
Exon 43	c.3181_3182delAG	p.(Ser1061Trpfs*2)	Frameshift	Haploinsufficient	Absent			PVS1 PS4 PM2(s) PP1	Pathogenic	Unknown	
Exon 44	c.3213_3221del	p.(Pro1072_Gly1074del)	In frame del	Splice/delins/del	Absent			PS4 PM2(s) PM4 PP1	Likely pathogenic	No	
Exon 44	c.3219_3222dup	p.(Ala1075Trpfs*20)	Frameshift	Haploinsufficient	Absent			PVS1 PS4 PM2(s) PP1	Pathogenic	Unknown	
Exon 46	c.3391G>A	p.(Gly1131Ser)	Missense	Glycine substitution	Absent	4/4	-	PS4 PM1 PM2(s) PP2 PP3 PP5	Pathogenic	Unknown	(5)
Exon 47	c.3446G>A	p.(Gly1149Asp)	Missense	Glycine substitution	Absent	4/4	-	PS4 PM1 PM2(s) PP2 PP3 PP5	Pathogenic	No	(1)
Exon 47	c.3512A>G	p.(Glu1171Gly)	Missense	Missense	Absent	4/4	-	PS4 PM2(s) PP2 PP3	Likely pathogenic	No	
Exon 48	c.3527_3528delinsAT	p.(Gly1176Asp)	Delins In frame	Splice/delins/del	Absent	3/3^	-	PS4 PM1 PM2(s) PM5 PP2 PP3	Pathogenic	Unknown	(5)
Exon 48	c.3653_3686delinsAATC	p.(pro1218_Ile1229delinsGlnSer)	Delins In frame	Splice/delins/del	Absent	-	-	PS4 PM2(s) PP1	Likely pathogenic	No	
	arr 2q31.3q32.3(180,458,048-194,965,339)x1, 15q13.3(29,8		CNV	Haploinsufficient				PSV1 PS4 PM6	Pathogenic	Yes	

	06,690-<br>30,211,011)x3										

NCBI Reference Sequence: NM_000090.3, NC_000002.12. gnomAD, Genome Aggregation Database v2.1.1; MAF, minor allele frequency; Ref, reference

† Based on MutationTaster, PolyPhen2, SIFT, align GVGD

PVS1: Probability of loss of function intolerance (pLI) score for COL3A1 is 1.0; loss of function observed/expected upper bound fraction (LOEUF) score for COL3A1 is 0.098. PVS1 is used as a moderate argument if RNA sequencing was not performed.

Δ PS4 is given after calculating Bayesian OR according to the recommendation of Kyung Cho et al [55]. Odds ratio are > 5.0 and confidence interval does not include 1.0.

ϴ PM1 Variant occurs in the triple helical domain and replaces the glycine in the canonical Gly-X-Y repeat; missense substitution of a canonical glycine residue is expected to disrupt normal protein folding and function, and this is an established mechanism of disease

T PM2 is used as a supportive argument according to the latest recommendations; ClinGen Sequence Variant Interpretation Recommendation for PM2 - Version 1.0

Y Specification of PP2; The missense constraint score (Z score ≥3.09) from gnomAD is used [56, 57]. Z-score for COL3A1 is 4.09 (https://gnomad.broadinstitute.org), the missense variants are spread over all exons.

˜ PP5 is given when a variant is previously described as P/LP in literature or in ClinVar.

*Suspected based on prediction models in Alamut.

V Based on described protein change by Pepin et al. [5].

^ MutationTaster not applicable

Supplemental Table 4. Specification thoracic aortic aneurysms

Patient	Sex	Mutation	Age at diagnosis (years)	Diameter thoracic aortic aneurysm (mm)	Imaging modality	Elective aortic surgery
**Ascending aortic aneurysm (n=10)**						
1	Male	p.(Gly252Valfs*11)	55	41	MRA	-
2	Male	p.(Gly342Arg)	63	45	Echocardiography	-
3	Female	c.(79+1_80-1)_(447+1_448-1)del.	56	53	n.a.	PEARS
4	Male	p.(Gly1149Asp)	41	47	CTA	Ascending aortic replacement (unspecified)
5	Female	p.(Gly1149Asp)	70	46	CTA	Ascending aortic replacement (unspecified)
6	Female	p.(Gly345Glu)	72	41	CTA	-
7	Male	p.(Gly393Arg)	69	41	CTA	-
8	Male	p.(Gly345Glu)	55	52	CTA	AVR + ascending aorta replacement
9	Male	p.(Ser1061Trpfs*2)	62	53	CTA	AVR + ascending aorta + partial arch replacement
10	Male	p.(Gly186Valfs*36)	46	47	CTA	-
**Ascending aortic aneurysm + abdominal aortic aneurysm (n=3)**						
11	Female	p.(Gly345Glu)	61	46	MRA	AVR + ascending aorta replacement + iliac endovascular stent
12	Male	p.(Thr911Trpfs*14)	53	48	CTA	-
13	Female	p.(Gly219fs)	55	40	MRA	-
**Sinus of Vasalva aneurysm (n=2)**						
14	Male	p.(Gly798Val)	36	42	CTA	-
15	Male	p.(Gly345Glu)	33	41	MRA	-
**Aortic arch aneurysm (n=3)**						
16	Male	p.(Gly393Arg)	43	40	CTA	-
17	Male	p.(Gly363Asp)	41	56	CTA	Ascending aorta + (partial) arch replacement
18	Male	p.(Gly327Asp)	69	40	MRA	-

MRA, Magnetic resonance angiography; PEARS, personalized external aortic root support; CTA, computed tomography angiography; AVR, aortic valve replacement; n.a., not applicable

Supplemental Table 5. Vascular interventions

Thoracic aorta			
	**Elective aneurysm repair (n=16)**	**Type A dissection (n= 8)**	**Type B dissection (n=5)**
**Operation performed (% of total)**	8 (50%)	6 (75%)	1 (20%)
**Diameters in mm (if known)**	46, 46, 47, 52, 53, 56		
**Type of intervention (n)**			
Ascending aortic replacement (unspecified)	2	1	
Ascending aorta + (partial) arch replacement	1	3	
AVR + ascending aorta replacement	2	2	
AVR + ascending aorta + partial arch replacement	1		
PEARS	1		
Descending aorta replacement	1		
TEVAR			1
**Severe complication**			
Sternum infection		1	
Abdominal aorta			
	**Elective aneurysm repair (n=16)**	**Rupture AAA (n=1)**	**Dissection (n=10)**
**Operation performed (% of total)**	6 (37.5%)	1 (100%)	5 (50%)
**Diameters in mm (if known)**	30, 57, 64, 88		
**Type of intervention (n)**			
(F)EVAR procedure	3		
Aortic bifurcation prothesis	2	1	1
Aorto-bi-iliac bypass	1		
Coiling			
Stenting			4
**Severe complications**			
Platzbauch and enterocutaneous fistulas			1
Type 3 endoleak, resulting in ruptured AAA (see next column)	1		
Serosa defect leading to ileocecal resection		1	
Middle sized arteries			
	**Aneurysm (n=18)**	**Dissection (n=19)**	**CCSF (n=2)**
**Operation performed (% of total)**	11 (50%)	5 (52.6%)	2 (100%)
**Type of intervention (n)**			
Coiling	5		2
Stenting	3	4	
Ligation	1		
Graft		1	
Unknown	2		
**Severe complication**			
Rupture after coiling		1	

AVR, aortic valve replacement; PEARS, personalized external aortic root support; TEVAR, thoracic endovascular aortic repair; (F)EVAR, (fenestrated) endovascular aortic repair; AAA, abdominal aortic aneurysm; CCSF, carotid-cavernous sinus fistula
